# Supplementary material for: Framing the potential of public frameshift peptides as immunotherapy targets in colon cancer
Source: PLoS One. 2021 Jun 28;16(6):e0251630. doi: 10.1371/journal.pone.0251630 (PMC8238217; doi:10.1371/journal.pone.0251630)
Supplement: S1 Fig — Density map showing difference between Z-score and VAF-ratio approach for the measurement of Nonsense Mediated Decay (NMD) in NMD RES and SENS mutations. Separation of NMD RES and SENS is clearer with VAF-ratio. (DOCX) [file pone.0251630.s001.docx]

# Supplementary Figures


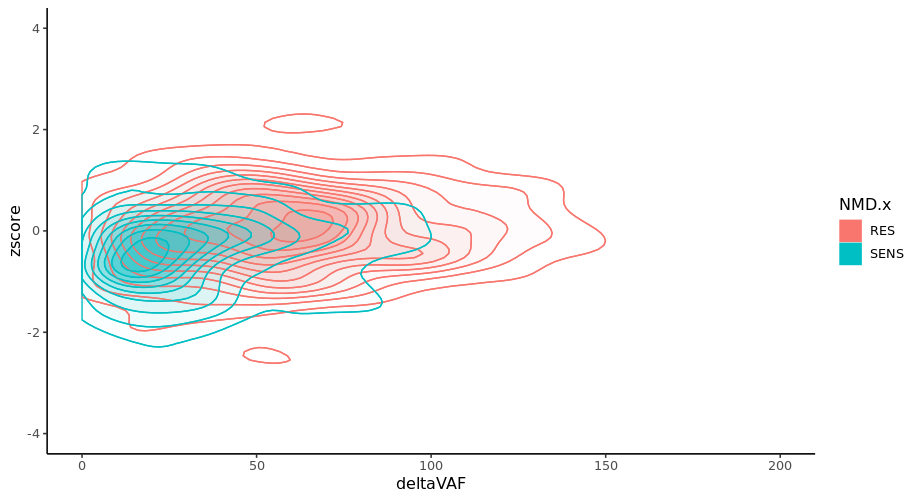

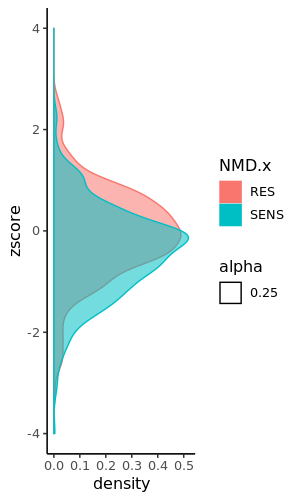

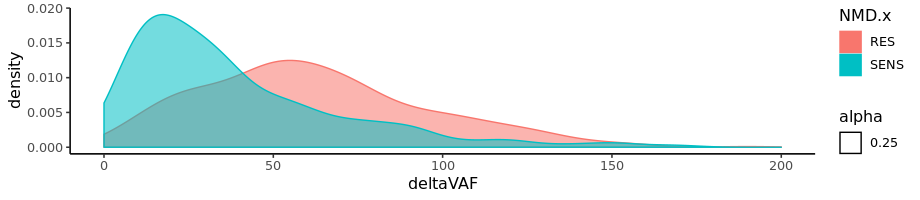

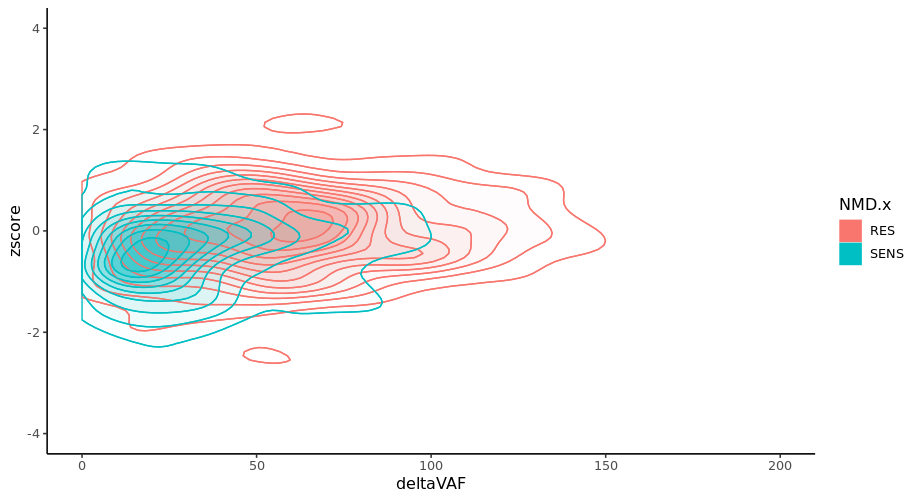


**VAF-ratio (%)**

**Supplementary figure 1. Difference in NMD measurement.** Density map showing difference between Z-score and VAF-ratio approach for the measurement of Nonsense Mediated Decay (NMD) in NMD RES and SENS mutations. Separation of NMD RES and SENS is clearer with VAF-ratio.
