# Supplementary material for: Framing the potential of public frameshift peptides as immunotherapy targets in colon cancer
Source: PLoS One. 2021 Jun 28;16(6):e0251630. doi: 10.1371/journal.pone.0251630 (PMC8238217; doi:10.1371/journal.pone.0251630)
Supplement: S2 Fig — PTC distance for mutation in MSI_H. Median is shown as dotted line (median = 14 Amino Acids). Dashed line shows 20 Amino Acids. (DOCX) [file pone.0251630.s002.docx]

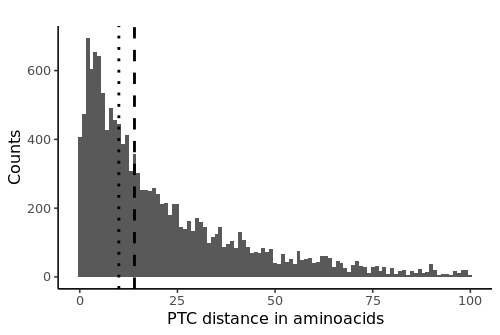


**Supplementary figure 2. Premature Termination Codon (PTC) distance.** PTC distance for mutation in MSI_H. Median is shown as dotted line (median = 14 Amino Acids ). Dashed line shows 20 Amino Acids.
