# Supplementary material for: Framing the potential of public frameshift peptides as immunotherapy targets in colon cancer
Source: PLoS One. 2021 Jun 28;16(6):e0251630. doi: 10.1371/journal.pone.0251630 (PMC8238217; doi:10.1371/journal.pone.0251630)
Supplement: S3 Fig — Percentage of patients covered by n most frequently occurring frameshift mutations with adequate expression, a SNORF and NMD-escape. (DOCX) [file pone.0251630.s003.docx]

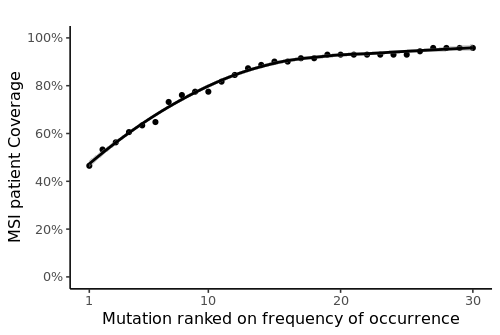


**Supplementary figure 3. Patient coverage for top 30 frameshift mutations.** Percentage of patients covered by n most frequently occurring frameshift mutations with adequate expression, a SNORF and NMD-escape.
