# Supplementary material for: Framing the potential of public frameshift peptides as immunotherapy targets in colon cancer
Source: PLoS One. 2021 Jun 28;16(6):e0251630. doi: 10.1371/journal.pone.0251630 (PMC8238217; doi:10.1371/journal.pone.0251630)
Supplement: S1 Table — List with genes described in the candidate cancer gene database that are part of the top 100 most occurring mutations in MSI colon cancer. Rank: Relative rank assigned to CIS in study. (DOCX) [file pone.0251630.s004.docx]

# Supplementary Tables

| Mouse Symbol | Mouse ID | Human Symbol | Cancer Type | Rank |
| --- | --- | --- | --- | --- |
| [Larp4b](http://ccgd-starrlab.oit.umn.edu/detail.php?gene_id=5058) | 217980 | LARP4B | Blood Cancer | B |
| [Larp4b](http://ccgd-starrlab.oit.umn.edu/detail.php?gene_id=5060) | 217980 | LARP4B | Breast Cancer | B |
| [Larp4b](http://ccgd-starrlab.oit.umn.edu/detail.php?gene_id=5045) | 217980 | LARP4B | Colorectal Cancer | A |
| [Larp4b](http://ccgd-starrlab.oit.umn.edu/detail.php?gene_id=5059) | 217980 | LARP4B | Colorectal Cancer | A |
| [Larp4b](http://ccgd-starrlab.oit.umn.edu/detail.php?gene_id=5055) | 217980 | LARP4B | Colorectal Cancer | C |
| [Larp4b](http://ccgd-starrlab.oit.umn.edu/detail.php?gene_id=5054) | 217980 | LARP4B | Colorectal Cancer | D |
| [Larp4b](http://ccgd-starrlab.oit.umn.edu/detail.php?gene_id=5056) | 217980 | LARP4B | Gastric Cancer | B |
| [Larp4b](http://ccgd-starrlab.oit.umn.edu/detail.php?gene_id=5052) | 217980 | LARP4B | Liver Cancer | A |
| [Larp4b](http://ccgd-starrlab.oit.umn.edu/detail.php?gene_id=5057) | 217980 | LARP4B | Liver Cancer | A |
| [Larp4b](http://ccgd-starrlab.oit.umn.edu/detail.php?gene_id=5043) | 217980 | LARP4B | Nervous System Cancer | C |
| [Larp4b](http://ccgd-starrlab.oit.umn.edu/detail.php?gene_id=5044) | 217980 | LARP4B | Nervous System Cancer | D |
| [Larp4b](http://ccgd-starrlab.oit.umn.edu/detail.php?gene_id=5049) | 217980 | LARP4B | Nervous System Cancer | D |
| [Larp4b](http://ccgd-starrlab.oit.umn.edu/detail.php?gene_id=5050) | 217980 | LARP4B | Nervous System Cancer | D |
| [Larp4b](http://ccgd-starrlab.oit.umn.edu/detail.php?gene_id=5051) | 217980 | LARP4B | Nervous System Cancer | Not Ranked |
| [Larp4b](http://ccgd-starrlab.oit.umn.edu/detail.php?gene_id=5046) | 217980 | LARP4B | Pancreatic Cancer | B |
| [Larp4b](http://ccgd-starrlab.oit.umn.edu/detail.php?gene_id=5047) | 217980 | LARP4B | Pancreatic Cancer | B |
| [Larp4b](http://ccgd-starrlab.oit.umn.edu/detail.php?gene_id=5048) | 217980 | LARP4B | Skin Cancer | A |
| [Larp4b](http://ccgd-starrlab.oit.umn.edu/detail.php?gene_id=5053) | 217980 | LARP4B | Skin Cancer | C |
| [Mecom](http://ccgd-starrlab.oit.umn.edu/detail.php?gene_id=11666) | 14013 | MECOM | Blood Cancer | A |
| [Mecom](http://ccgd-starrlab.oit.umn.edu/detail.php?gene_id=11671) | 14013 | MECOM | Blood Cancer | A |
| [Mecom](http://ccgd-starrlab.oit.umn.edu/detail.php?gene_id=11677) | 14013 | MECOM | Blood Cancer | A |
| [Mecom](http://ccgd-starrlab.oit.umn.edu/detail.php?gene_id=11674) | 14013 | MECOM | Blood Cancer | B |
| [Mecom](http://ccgd-starrlab.oit.umn.edu/detail.php?gene_id=11676) | 14013 | MECOM | Blood Cancer | B |
| [Mecom](http://ccgd-starrlab.oit.umn.edu/detail.php?gene_id=11675) | 14013 | MECOM | Blood Cancer | D |
| [Mecom](http://ccgd-starrlab.oit.umn.edu/detail.php?gene_id=11672) | 14013 | MECOM | Blood Cancer | Not Ranked |
| [Mecom](http://ccgd-starrlab.oit.umn.edu/detail.php?gene_id=11673) | 14013 | MECOM | Blood Cancer | Not Ranked |
| [Mecom](http://ccgd-starrlab.oit.umn.edu/detail.php?gene_id=11668) | 14013 | MECOM | Colorectal Cancer | A |
| [Mecom](http://ccgd-starrlab.oit.umn.edu/detail.php?gene_id=11670) | 14013 | MECOM | Colorectal Cancer | D |
| [Mecom](http://ccgd-starrlab.oit.umn.edu/detail.php?gene_id=11680) | 14013 | MECOM | Colorectal Cancer | D |
| [Mecom](http://ccgd-starrlab.oit.umn.edu/detail.php?gene_id=11679) | 14013 | MECOM | Gastric Cancer | A |
| [Mecom](http://ccgd-starrlab.oit.umn.edu/detail.php?gene_id=11667) | 14013 | MECOM | Pancreatic Cancer | C |
| [Mecom](http://ccgd-starrlab.oit.umn.edu/detail.php?gene_id=11669) | 14013 | MECOM | Pancreatic Cancer | C |
| [Mecom](http://ccgd-starrlab.oit.umn.edu/detail.php?gene_id=11678) | 14013 | MECOM | Pancreatic Cancer | D |
| [Acvr2a](http://ccgd-starrlab.oit.umn.edu/detail.php?gene_id=21107) | 11480 | ACVR2A | Breast Cancer | C |
| [Acvr2a](http://ccgd-starrlab.oit.umn.edu/detail.php?gene_id=21101) | 11480 | ACVR2A | Colorectal Cancer | A |
| [Acvr2a](http://ccgd-starrlab.oit.umn.edu/detail.php?gene_id=21106) | 11480 | ACVR2A | Colorectal Cancer | B |
| [Acvr2a](http://ccgd-starrlab.oit.umn.edu/detail.php?gene_id=21097) | 11480 | ACVR2A | Colorectal Cancer | C |
| [Acvr2a](http://ccgd-starrlab.oit.umn.edu/detail.php?gene_id=21103) | 11480 | ACVR2A | Colorectal Cancer | C |
| [Acvr2a](http://ccgd-starrlab.oit.umn.edu/detail.php?gene_id=21102) | 11480 | ACVR2A | Colorectal Cancer | D |
| [Acvr2a](http://ccgd-starrlab.oit.umn.edu/detail.php?gene_id=21104) | 11480 | ACVR2A | Gastric Cancer | C |
| [Acvr2a](http://ccgd-starrlab.oit.umn.edu/detail.php?gene_id=21100) | 11480 | ACVR2A | Liver Cancer | C |
| [Acvr2a](http://ccgd-starrlab.oit.umn.edu/detail.php?gene_id=21105) | 11480 | ACVR2A | Liver Cancer | C |
| [Acvr2a](http://ccgd-starrlab.oit.umn.edu/detail.php?gene_id=21095) | 11480 | ACVR2A | Mixed | Not Ranked |
| [Acvr2a](http://ccgd-starrlab.oit.umn.edu/detail.php?gene_id=21099) | 11480 | ACVR2A | Pancreatic Cancer | A |
| [Acvr2a](http://ccgd-starrlab.oit.umn.edu/detail.php?gene_id=21096) | 11480 | ACVR2A | Pancreatic Cancer | B |
| [Acvr2a](http://ccgd-starrlab.oit.umn.edu/detail.php?gene_id=21098) | 11480 | ACVR2A | Pancreatic Cancer | B |
| [Qk](http://ccgd-starrlab.oit.umn.edu/detail.php?gene_id=21419) | 19317 | QKI | Blood Cancer | A |
| [Qk](http://ccgd-starrlab.oit.umn.edu/detail.php?gene_id=21417) | 19317 | QKI | Gastric Cancer | B |
| [Qk](http://ccgd-starrlab.oit.umn.edu/detail.php?gene_id=21413) | 19317 | QKI | Liver Cancer | A |
| [Qk](http://ccgd-starrlab.oit.umn.edu/detail.php?gene_id=21418) | 19317 | QKI | Liver Cancer | A |
| [Qk](http://ccgd-starrlab.oit.umn.edu/detail.php?gene_id=21408) | 19317 | QKI | Liver Cancer | B |
| [Qk](http://ccgd-starrlab.oit.umn.edu/detail.php?gene_id=21407) | 19317 | QKI | Liver Cancer | C |
| [Qk](http://ccgd-starrlab.oit.umn.edu/detail.php?gene_id=21411) | 19317 | QKI | Nervous System Cancer | A |
| [Qk](http://ccgd-starrlab.oit.umn.edu/detail.php?gene_id=21412) | 19317 | QKI | Nervous System Cancer | A |
| [Qk](http://ccgd-starrlab.oit.umn.edu/detail.php?gene_id=21409) | 19317 | QKI | Nervous System Cancer | B |
| [Qk](http://ccgd-starrlab.oit.umn.edu/detail.php?gene_id=21410) | 19317 | QKI | Nervous System Cancer | B |
| [Qk](http://ccgd-starrlab.oit.umn.edu/detail.php?gene_id=21414) | 19317 | QKI | Pancreatic Cancer | B |
| [Qk](http://ccgd-starrlab.oit.umn.edu/detail.php?gene_id=21416) | 19317 | QKI | Sarcoma | A |
| [Qk](http://ccgd-starrlab.oit.umn.edu/detail.php?gene_id=21415) | 19317 | QKI | Sarcoma | C |
| [Zbtb20](http://ccgd-starrlab.oit.umn.edu/detail.php?gene_id=21523) | 56490 | ZBTB20 | Blood Cancer | A |
| [Zbtb20](http://ccgd-starrlab.oit.umn.edu/detail.php?gene_id=21512) | 56490 | ZBTB20 | Liver Cancer | A |
| [Zbtb20](http://ccgd-starrlab.oit.umn.edu/detail.php?gene_id=21511) | 56490 | ZBTB20 | Liver Cancer | B |
| [Zbtb20](http://ccgd-starrlab.oit.umn.edu/detail.php?gene_id=21519) | 56490 | ZBTB20 | Liver Cancer | B |
| [Zbtb20](http://ccgd-starrlab.oit.umn.edu/detail.php?gene_id=21522) | 56490 | ZBTB20 | Liver Cancer | B |
| [Zbtb20](http://ccgd-starrlab.oit.umn.edu/detail.php?gene_id=21516) | 56490 | ZBTB20 | Nervous System Cancer | A |
| [Zbtb20](http://ccgd-starrlab.oit.umn.edu/detail.php?gene_id=21517) | 56490 | ZBTB20 | Nervous System Cancer | A |
| [Zbtb20](http://ccgd-starrlab.oit.umn.edu/detail.php?gene_id=21513) | 56490 | ZBTB20 | Nervous System Cancer | D |
| [Zbtb20](http://ccgd-starrlab.oit.umn.edu/detail.php?gene_id=21520) | 56490 | ZBTB20 | Nervous System Cancer | D |
| [Zbtb20](http://ccgd-starrlab.oit.umn.edu/detail.php?gene_id=21518) | 56490 | ZBTB20 | Nervous System Cancer | Not Ranked |
| [Zbtb20](http://ccgd-starrlab.oit.umn.edu/detail.php?gene_id=21514) | 56490 | ZBTB20 | Pancreatic Cancer | A |
| [Zbtb20](http://ccgd-starrlab.oit.umn.edu/detail.php?gene_id=21515) | 56490 | ZBTB20 | Pancreatic Cancer | B |
| [Zbtb20](http://ccgd-starrlab.oit.umn.edu/detail.php?gene_id=21521) | 56490 | ZBTB20 | Skin Cancer | C |
| [Son](http://ccgd-starrlab.oit.umn.edu/detail.php?gene_id=25473) | 20658 | SON | Blood Cancer | D |
| [Son](http://ccgd-starrlab.oit.umn.edu/detail.php?gene_id=25475) | 20658 | SON | Breast Cancer | D |
| [Son](http://ccgd-starrlab.oit.umn.edu/detail.php?gene_id=25464) | 20658 | SON | Colorectal Cancer | B |
| [Son](http://ccgd-starrlab.oit.umn.edu/detail.php?gene_id=25470) | 20658 | SON | Colorectal Cancer | C |
| [Son](http://ccgd-starrlab.oit.umn.edu/detail.php?gene_id=25474) | 20658 | SON | Colorectal Cancer | D |
| [Son](http://ccgd-starrlab.oit.umn.edu/detail.php?gene_id=25471) | 20658 | SON | Gastric Cancer | D |
| [Son](http://ccgd-starrlab.oit.umn.edu/detail.php?gene_id=25469) | 20658 | SON | Liver Cancer | A |
| [Son](http://ccgd-starrlab.oit.umn.edu/detail.php?gene_id=25472) | 20658 | SON | Liver Cancer | A |
| [Son](http://ccgd-starrlab.oit.umn.edu/detail.php?gene_id=25467) | 20658 | SON | Nervous System Cancer | D |
| [Son](http://ccgd-starrlab.oit.umn.edu/detail.php?gene_id=25468) | 20658 | SON | Nervous System Cancer | D |
| [Son](http://ccgd-starrlab.oit.umn.edu/detail.php?gene_id=25466) | 20658 | SON | Pancreatic Cancer | C |
| [Son](http://ccgd-starrlab.oit.umn.edu/detail.php?gene_id=25465) | 20658 | SON | Pancreatic Cancer | D |
| [Rnf43](http://ccgd-starrlab.oit.umn.edu/detail.php?gene_id=37693) | 207742 | RNF43 | Blood Cancer | B |
| [Rnf43](http://ccgd-starrlab.oit.umn.edu/detail.php?gene_id=37694) | 207742 | RNF43 | Colorectal Cancer | A |
| [Rnf43](http://ccgd-starrlab.oit.umn.edu/detail.php?gene_id=37685) | 207742 | RNF43 | Colorectal Cancer | B |
| [Rnf43](http://ccgd-starrlab.oit.umn.edu/detail.php?gene_id=37689) | 207742 | RNF43 | Colorectal Cancer | B |
| [Rnf43](http://ccgd-starrlab.oit.umn.edu/detail.php?gene_id=37690) | 207742 | RNF43 | Colorectal Cancer | D |
| [Rnf43](http://ccgd-starrlab.oit.umn.edu/detail.php?gene_id=37691) | 207742 | RNF43 | Gastric Cancer | A |
| [Rnf43](http://ccgd-starrlab.oit.umn.edu/detail.php?gene_id=37688) | 207742 | RNF43 | Liver Cancer | D |
| [Rnf43](http://ccgd-starrlab.oit.umn.edu/detail.php?gene_id=37692) | 207742 | RNF43 | Liver Cancer | D |
| [Rnf43](http://ccgd-starrlab.oit.umn.edu/detail.php?gene_id=37687) | 207742 | RNF43 | Pancreatic Cancer | A |
| [Rnf43](http://ccgd-starrlab.oit.umn.edu/detail.php?gene_id=37686) | 207742 | RNF43 | Pancreatic Cancer | B |
| [Tnrc6c](http://ccgd-starrlab.oit.umn.edu/detail.php?gene_id=44673) | 217351 | TNRC6C | Blood Cancer | A |
| [Tnrc6c](http://ccgd-starrlab.oit.umn.edu/detail.php?gene_id=44665) | 217351 | TNRC6C | Colorectal Cancer | C |
| [Tnrc6c](http://ccgd-starrlab.oit.umn.edu/detail.php?gene_id=44670) | 217351 | TNRC6C | Liver Cancer | D |
| [Tnrc6c](http://ccgd-starrlab.oit.umn.edu/detail.php?gene_id=44672) | 217351 | TNRC6C | Liver Cancer | D |
| [Tnrc6c](http://ccgd-starrlab.oit.umn.edu/detail.php?gene_id=44668) | 217351 | TNRC6C | Nervous System Cancer | D |
| [Tnrc6c](http://ccgd-starrlab.oit.umn.edu/detail.php?gene_id=44669) | 217351 | TNRC6C | Nervous System Cancer | D |
| [Tnrc6c](http://ccgd-starrlab.oit.umn.edu/detail.php?gene_id=44666) | 217351 | TNRC6C | Pancreatic Cancer | C |
| [Tnrc6c](http://ccgd-starrlab.oit.umn.edu/detail.php?gene_id=44667) | 217351 | TNRC6C | Pancreatic Cancer | D |
| [Tnrc6c](http://ccgd-starrlab.oit.umn.edu/detail.php?gene_id=44671) | 217351 | TNRC6C | Sarcoma | B |
| [Smarcad1](http://ccgd-starrlab.oit.umn.edu/detail.php?gene_id=72229) | 13990 | SMARCAD1 | Blood Cancer | D |
| [Smarcad1](http://ccgd-starrlab.oit.umn.edu/detail.php?gene_id=72226) | 13990 | SMARCAD1 | Colorectal Cancer | B |
| [Smarcad1](http://ccgd-starrlab.oit.umn.edu/detail.php?gene_id=72227) | 13990 | SMARCAD1 | Colorectal Cancer | D |
| [Smarcad1](http://ccgd-starrlab.oit.umn.edu/detail.php?gene_id=72230) | 13990 | SMARCAD1 | Colorectal Cancer | D |
| [Smarcad1](http://ccgd-starrlab.oit.umn.edu/detail.php?gene_id=72225) | 13990 | SMARCAD1 | Pancreatic Cancer | D |
| [Smarcad1](http://ccgd-starrlab.oit.umn.edu/detail.php?gene_id=72228) | 13990 | SMARCAD1 | Sarcoma | D |
| [Tmpo](http://ccgd-starrlab.oit.umn.edu/detail.php?gene_id=72361) | 21917 | TMPO | Blood Cancer | C |
| [Tmpo](http://ccgd-starrlab.oit.umn.edu/detail.php?gene_id=72359) | 21917 | TMPO | Colorectal Cancer | B |
| [Tmpo](http://ccgd-starrlab.oit.umn.edu/detail.php?gene_id=72362) | 21917 | TMPO | Colorectal Cancer | C |
| [Tmpo](http://ccgd-starrlab.oit.umn.edu/detail.php?gene_id=72357) | 21917 | TMPO | Colorectal Cancer | D |
| [Tmpo](http://ccgd-starrlab.oit.umn.edu/detail.php?gene_id=72358) | 21917 | TMPO | Liver Cancer | C |
| [Tmpo](http://ccgd-starrlab.oit.umn.edu/detail.php?gene_id=72360) | 21917 | TMPO | Liver Cancer | D |
| [Csmd3](http://ccgd-starrlab.oit.umn.edu/detail.php?gene_id=83403) | 239420 | CSMD3 | Gastric Cancer | D |
| [Csmd3](http://ccgd-starrlab.oit.umn.edu/detail.php?gene_id=83404) | 239420 | CSMD3 | Liver Cancer | A |
| [Csmd3](http://ccgd-starrlab.oit.umn.edu/detail.php?gene_id=83401) | 239420 | CSMD3 | Mixed | Not Ranked |
| [Csmd3](http://ccgd-starrlab.oit.umn.edu/detail.php?gene_id=83402) | 239420 | CSMD3 | Nervous System Cancer | Not Ranked |
| [Csmd3](http://ccgd-starrlab.oit.umn.edu/detail.php?gene_id=83400) | 239420 | CSMD3 | Sarcoma | A |
| [Bmpr2](http://ccgd-starrlab.oit.umn.edu/detail.php?gene_id=98735) | 12168 | BMPR2 | Blood Cancer | B |
| [Bmpr2](http://ccgd-starrlab.oit.umn.edu/detail.php?gene_id=98734) | 12168 | BMPR2 | Gastric Cancer | C |
| [Bmpr2](http://ccgd-starrlab.oit.umn.edu/detail.php?gene_id=98733) | 12168 | BMPR2 | Nervous System Cancer | B |
| [Bmpr2](http://ccgd-starrlab.oit.umn.edu/detail.php?gene_id=98732) | 12168 | BMPR2 | Nervous System Cancer | C |
| [Brd3](http://ccgd-starrlab.oit.umn.edu/detail.php?gene_id=98745) | 67382 | BRD3 | Blood Cancer | A |
| [Brd3](http://ccgd-starrlab.oit.umn.edu/detail.php?gene_id=98746) | 67382 | BRD3 | Liver Cancer | D |
| [Brd3](http://ccgd-starrlab.oit.umn.edu/detail.php?gene_id=98747) | 67382 | BRD3 | Liver Cancer | D |
| [Brd3](http://ccgd-starrlab.oit.umn.edu/detail.php?gene_id=98744) | 67382 | BRD3 | Pancreatic Cancer | D |
| [Slc3a2](http://ccgd-starrlab.oit.umn.edu/detail.php?gene_id=100149) | 17254 | SLC3A2 | Blood Cancer | B |
| [Slc3a2](http://ccgd-starrlab.oit.umn.edu/detail.php?gene_id=100150) | 17254 | SLC3A2 | Colorectal Cancer | D |
| [Slc3a2](http://ccgd-starrlab.oit.umn.edu/detail.php?gene_id=100148) | 17254 | SLC3A2 | Colorectal Cancer | Not Ranked |
| [Slc3a2](http://ccgd-starrlab.oit.umn.edu/detail.php?gene_id=100151) | 17254 | SLC3A2 | Liver Cancer | Not Ranked |
| [Svil](http://ccgd-starrlab.oit.umn.edu/detail.php?gene_id=100265) | 225115 | SVIL | Colorectal Cancer | D |
| [Svil](http://ccgd-starrlab.oit.umn.edu/detail.php?gene_id=100267) | 225115 | SVIL | Gastric Cancer | D |
| [Svil](http://ccgd-starrlab.oit.umn.edu/detail.php?gene_id=100264) | 225115 | SVIL | Liver Cancer | D |
| [Svil](http://ccgd-starrlab.oit.umn.edu/detail.php?gene_id=100266) | 225115 | SVIL | Lung Cancer | D |
| [Hnrnpl](http://ccgd-starrlab.oit.umn.edu/detail.php?gene_id=118097) | 15388 | HNRNPL | Colorectal Cancer | Not Ranked |
| [Hnrnpl](http://ccgd-starrlab.oit.umn.edu/detail.php?gene_id=118098) | 15388 | HNRNPL | Liver Cancer | D |
| [Hnrnpl](http://ccgd-starrlab.oit.umn.edu/detail.php?gene_id=118099) | 15388 | HNRNPL | Liver Cancer | D |
| [Nap1l1](http://ccgd-starrlab.oit.umn.edu/detail.php?gene_id=118525) | 53605 | NAP1L1 | Blood Cancer | D |
| [Nap1l1](http://ccgd-starrlab.oit.umn.edu/detail.php?gene_id=118524) | 53605 | NAP1L1 | Gastric Cancer | D |
| [Nap1l1](http://ccgd-starrlab.oit.umn.edu/detail.php?gene_id=118523) | 53605 | NAP1L1 | Pancreatic Cancer | A |
| [Wdtc1](http://ccgd-starrlab.oit.umn.edu/detail.php?gene_id=119731) | 230796 | WDTC1 | Blood Cancer | B |
| [Wdtc1](http://ccgd-starrlab.oit.umn.edu/detail.php?gene_id=119729) | 230796 | WDTC1 | Colorectal Cancer | Not Ranked |
| [Wdtc1](http://ccgd-starrlab.oit.umn.edu/detail.php?gene_id=119730) | 230796 | WDTC1 | Colorectal Cancer | Not Ranked |
| [Camta2](http://ccgd-starrlab.oit.umn.edu/detail.php?gene_id=142870) | 216874 | CAMTA2 | Blood Cancer | D |
| [Camta2](http://ccgd-starrlab.oit.umn.edu/detail.php?gene_id=142869) | 216874 | CAMTA2 | Colorectal Cancer | Not Ranked |
| [Plekha6](http://ccgd-starrlab.oit.umn.edu/detail.php?gene_id=144984) | 240753 | PLEKHA6 | Colorectal Cancer | D |
| [Plekha6](http://ccgd-starrlab.oit.umn.edu/detail.php?gene_id=144983) | 240753 | PLEKHA6 | Gastric Cancer | D |
| [Card11](http://ccgd-starrlab.oit.umn.edu/detail.php?gene_id=177209) | 108723 | CARD11 | Blood Cancer | C |
| [Ccr5](http://ccgd-starrlab.oit.umn.edu/detail.php?gene_id=177301) | 12774 | CCR5 | Blood Cancer | C |
| [Cemip2](http://ccgd-starrlab.oit.umn.edu/detail.php?gene_id=177379) | 83921 | CEMIP2 | Blood Cancer | B |
| [Dock3](http://ccgd-starrlab.oit.umn.edu/detail.php?gene_id=177795) | 208869 | DOCK3 | Blood Cancer | B |
| [Fhod3](http://ccgd-starrlab.oit.umn.edu/detail.php?gene_id=178107) | 225288 | FHOD3 | Blood Cancer | D |
| [Igf2r](http://ccgd-starrlab.oit.umn.edu/detail.php?gene_id=178578) | 16004 | IGF2R | Nervous System Cancer | Not Ranked |
| [Nr1h2](http://ccgd-starrlab.oit.umn.edu/detail.php?gene_id=179371) | 22260 | NR1H2 | Blood Cancer | D |
| [Rab28](http://ccgd-starrlab.oit.umn.edu/detail.php?gene_id=179926) | 100972 | RAB28 | Blood Cancer | D |
| [Rpl22](http://ccgd-starrlab.oit.umn.edu/detail.php?gene_id=180104) | 19934 | RPL22 | Liver Cancer | D |
| [Xylt2](http://ccgd-starrlab.oit.umn.edu/detail.php?gene_id=181177) | 217119 | XYLT2 | Blood Cancer | D |

**Supplementary Table 1. Candidate cancer genes .** List with genes described in the candidate cancer gene database that are part of the top 100 most occurring mutations in MSI colon cancer. Rank: Relative rank assigned to CIS in study
