# Supplementary material for: Framing the potential of public frameshift peptides as immunotherapy targets in colon cancer
Source: PLoS One. 2021 Jun 28;16(6):e0251630. doi: 10.1371/journal.pone.0251630 (PMC8238217; doi:10.1371/journal.pone.0251630)
Supplement: Attachment — Submitted filename: Plos One Reviewer response rebuttal.pdf [file pone.0251630.s005.pdf]

Amsterdam UMC
Universitair Medische Centra

Dr. Joerg Herber (editor-in-chief)
Editorial office
Plos One

AMC, Meibergdreef 9, Amsterdam
Medical Oncology
I.T. Spaanderman M.D.

Dear Editor,

We are very thankful for the positive feedback on our answers to the reviewer questions concerning our manuscript entitled “Framing the potential of public frameshift peptides as immunotherapy targets in colon cancer”. We are welcoming the final reviewers remarks to clarify the last outstanding issues. Attached you can find our response to these remarks and an improved version of our manuscript. We are confident that the alterations and additional analysis further clarify the main findings of our manuscript and therefore we sincerely hope you find our manuscript in its current form suitable for publication in PLOS ONE.

We are looking forward to hearing from you.

Yours Sincerely,

Ide T. Spaanderman MD, PhD candidate
Dept. of Experimental Immunology and Dept. Clinical Oncology
Amsterdam UMC

Attachments:

1. Response to reviewer questions
2. Updated manuscript

Response to reviewer questions

<u>Final remarks</u>

Reviewer #2 had no more comments. All comments below are concerning reviewer 1.

1. **Clarification of Zscore**
    I. They should consider discussing the caveats of this approach better in the text. Probably as they say this would work fine for the copy number alterations, as the patients are all MSI and from the same cancer types. But it might be less appropriate with respect to the gene expression variation between individuals, for instance if the patients are from distinct gene expression clusters/subtypes within the cancer (I do appreciate however that MSI is typically considered a single subtype within a given cancer type). Moreover, the results are compared between three cancer types, and the global tissue-specific gene expression variation is not accounted for.
        - First off al we thank reviewer 1 for this question and have tried to clarify the use of the Zscore within the text, stating that as suggested by the reviewer, this method is only suitable if used within a single population with the same molecular cancer subtype, as we did in our analysis, to avoid problems with copy number variation and gene expression variation.
2. **POLE MSS patients**
    I. Could the authors comment on the outliers in the MSS group in Fig 1 (TMB ~15000-20000) – are these potentially misclassified as MSS by TCGA? A comment to explain this should be added to the methods.We thank reviewer #1 for pointing out that our explanation of the Zscore and VAF-ratio concepts are insufficient in the manuscript. The transcript independent method is actually the VAF-ratio, where a lower VAF ratio indicates NMD. We have edited both the main text, the legend of figure 3 and the methods section to better reflect the above. As stated below the NMD classification has been replaced by NMDetective algoritm by Lindenboom et al. (*PMID: 31659324)* Considering the downregulation of NMD resistant mutation this is also a good questions, and we are thankfull that the reviewer made us realize that the explanation for this fact does not become very clear. Most likely this downregulation is due to the effect of the VAF-ratio method, that relies on the difference between the DNA and RNA VAF. The fact that DNA contains two alleles, whereas just one is copied to the RNA it becomes expected that even in the situation of NMD resistant genes the mean VAF-ratio is approximately 50%. We have added a clarification of this in the methods section.
        - We agree that with reviewer 1 that merely hinting at POLE for these patients is not sufficient. Therefore we have looked in detail at the mutational profile of these patients and concluded that these are indeed POLE patients. We have stated the exact muations in the text.
3. **SNORFS as vaccine**
    I. With respect to using those SNORFS as a potential vaccine - would it make sense to consider substituting some of the NMD-SENS predicted from the top 20 SNORFS, by other NMD-RES or with a higher stringent deltaVAF threshold, even though they are less frequent?

AMC en VUmc werken
samen in Amsterdam UMC

AMC
Meibergdreef 9
1105 AZ Amsterdam

Postbus 22660
1100 DD Amsterdam

T +31(0)20 566 9111
www.amc.nl

- We see reviewer 1 has a valid point regarding the selection of the the SNORFS, but we believe that selecting on higher deltaVAF thresholds only makes sense for vaccination in a personalized vaccination strategy. In our aim for a single vaccination for a single cancer subgroup we aim to select more frequently occurring mutations with a sufficient deltaVAF over less frequently occurring mutations with a higher deltaVAF. We have tried to clarify this in the discussion.

4. **NMD-inducing features of SNORFS**

   I. As a consideration for future work and/or something to mention in the Discussion, they may consider checking if these SNORFS have NMD-inducing features of NMD endogenous targets (~5-20% of genes), some of which are different from the PTC position rules: 5' upstream ORF, intron in 3' UTR, long 3' UTR etc.

   - We thank reviewer 1 for this suggestion. This seems to be a very interesting avenue of further research and we are considering implementing it in our new projects. Nonetheless we feel that it might further complicate our current manuscript and therefore decided not to include it.

5. **VAFratio calculation**

   I. There may be issues with clarity of descriptions of the VAFratio method. In methods, it would help if they could show the formula used for VAFratio calculation along with some examples (one NMD-sensitive and one NMD-resistant). It probably does not make much sense to show the z-score formula (which they finally don't seem to use) while not showing the VAFratio formula. From the current description, one could infer that the VAF-ratio is higher for NMD-sensitive transcripts than for NMD-resistant transcripts, but they state: "The transcript independent method is actually the VAF-ratio, where a lower VAF ratio indicates NMD", which seems to be suggesting the opposite. Please take care to clarify this.

   Also, "the RNA VAF only relies on the single allele that has been translated," and in the revisions-paragraph: "The fact that DNA contains two alleles, whereas just one is copied to the RNA" may be misleading, since both alleles are likely transcribed (while one may be degraded and the other no).We thank reviewer 1 for this suggestion. This seems to be a very interesting avenue of further research and we are considering implementing it in our new projects. Nonetheless we feel that it might further complicate our current manuscript and therefore decided not to include it.

   - Thanks to reviewer 1 we have realized that the VAFratio can be cause for some confusion when reading our manuscript. We have tried to clarify our use by two measures. First off all, as suggested by reviewer 1, we have added the VAFratio formula to the methods section of the manuscript, including examples of NMD escaping and non-escaping transcripts. Furthermore we have altered figure 3b in order to not create confusion about the signs of NMD in the NMD resistant transcripts, by normalizing the data to the NMD resistant mutations. The prior decrease in NMD resistant mutations is probably caused by many factors including the inaccuracy of the NMD prediction algorithm that is based on mRNA transcript level information instead of mutation based mRNA information and therefore is

AMC en VUmc werken samen in Amsterdam UMC

AMC
Meibergdreef 9
1105 AZ Amsterdam

Postbus 22660
1100 DD Amsterdam

T +31(0)20 566 9111
www.amc.nl

prone to missing exon skipping for example. We thinks this approach clarifies are underlying message that NMD sensitive transcripts are degraded more often, but that there is a substantial portion of mutated transcripts that does not get degraded by NMD and therefore can yield neo-epitopes.

AMC en VUmc werken
samen in Amsterdam UMC

AMC
Meibergdreef 9
1105 AZ Amsterdam

Postbus 22660
1100 DD Amsterdam

T +31(0)20 566 9111
www.amc.nl
